# Modelling collateral flow and thrombus permeability during acute ischaemic stroke

**DOI:** 10.1098/rsif.2022.0649

**Published:** 2022-10-05

**Authors:** Raymond M. Padmos, Nerea Arrarte Terreros, Tamás I. Józsa, Gábor Závodszky, Henk A. Marquering, Charles B. L. M. Majoie, Stephen J. Payne, Alfons G. Hoekstra

**Affiliations:** ^1^ Computational Science Laboratory, Informatics Institute, Faculty of Science, University of Amsterdam, Science Park 904, Amsterdam 1098, The Netherlands; ^2^ Faculty of Mechanical, Maritime and Materials Engineering, Delft University of Technology, Mekelweg 2, Delft 2628, The Netherlands; ^3^ Department of Radiology and Nuclear Medicine, Amsterdam UMC, location AMC, Amsterdam, The Netherlands; ^4^ Department of Biomedical Engineering and Physics, Amsterdam UMC, location AMC, Amsterdam, The Netherlands; ^5^ Institute of Biomedical Engineering, Department of Engineering Science, University of Oxford, Parks Road, Oxford OX1 3PJ, UK; ^6^ Department of Radiology and Nuclear Medicine, Amsterdam Neuroscience, Amsterdam University Medical Center, Location VUmc, Amsterdam, The Netherlands; ^7^ Institute of Applied Mechanics, National Taiwan University, Taiwan

**Keywords:** collateral flow, thrombus permeability, infarct volume, acute ischaemic stroke, blood flow modelling

## Abstract

The presence of collaterals and high thrombus permeability are associated with good functional outcomes after an acute ischaemic stroke. We aim to understand the combined effect of the collaterals and thrombus permeability on cerebral blood flow during an acute ischaemic stroke. A cerebral blood flow model including the leptomeningeal collateral circulation is used to simulate cerebral blood flow during an acute ischaemic stroke. The collateral circulation is varied to capture the collateral scores: absent, poor, moderate and good. Measurements of the transit time, void fraction and thrombus length in acute ischaemic stroke patients are used to estimate thrombus permeability. Estimated thrombus permeability ranges between 10^−7^ and 10^−4^ mm^2^. Measured flow rates through the thrombus are small and the effect of a permeable thrombus on brain perfusion during stroke is small compared with the effect of collaterals. Our simulations suggest that the collaterals are a dominant factor in the resulting infarct volume after a stroke.

## 1. Introduction

Acute ischaemic stroke (AIS) is one of the leading causes of death and disability worldwide [1]. AIS occurs when a thrombus occludes a major cerebral vessel, thereby restricting blood flow and starving downstream tissues of nutrients such as glucose and oxygen. High thrombus permeability and collateral score are two factors associated with good functional outcome in patients [2,3].

Collaterals are alternative pathways for blood flow to reach downstream tissue during AIS. The primary collateral pathway is the circle of Willis. Secondary pathways include, among others, the leptomeningeal collateral vessels on the pial surface [4]. The flow through the collaterals varies significantly among patients [5]. The variation in collateral flow is commonly captured in a collateral score, where the collaterals are graded as absent, poor, moderate or good based on the contrast filling of distal tissue [6]. The different collateral scores are defined as 0%, greater than 0% to less than or equal to 50%, greater than 50% to less than 100%, and 100% filling of the tissue with contrast agent, respectively, and graded visually [7].

Thrombus permeability is not measurable directly in AIS patients. Instead, a derived measure is often used; thrombus perviousness is defined as the ability of contrast to penetrate a thrombus [8]. A permeable thrombus would enable tissue to survive longer by allowing nutrients to reach downstream tissue. In addition, thrombus permeability has an important effect on intravenous treatment with tissue-type plasminogen activator (tPA) [9]. Without permeation, thrombolysis would be diffusion limited and require hundreds of minutes [10]. Given that thrombolysis is often an effective clinical treatment, there has to be some flow to the thrombus [11]. High perviousness is associated with better patient outcome even when patients do not receive tPA treatment [9].

The combined effect of thrombus permeability and collateral flow on remaining blood flow during AIS is not well understood. Thrombi in large cerebral blood flow models are often assumed to be completely occluding the vessel [12,13]. However, flow through the thrombus can be a significant factor for patient outcomes, especially if collateral flow is poor [9,14]. Understanding the effect of collaterals and thrombus permeability on residual blood flow to downstream tissue can improve patient outcome estimates, infarct volume estimation and treatment effectiveness after an AIS.

In this study, cerebral blood flow and pressure are simulated during an AIS using a one-dimensional blood flow (BF) model of the cerebral vasculature. The variation in number of leptomeningeal collateral vessels is included in the model. The model parameter defining the collaterals is calibrated to obtain similar infarct volumes for the various collateral scores as observed in patients. A permeable thrombus is modelled as a porous medium with a predefined permeability. A wide range of permeability and thrombus lengths are simulated using the one-dimensional BF model. The model is then used to estimate thrombus permeability, pressure drop and infarct volume using flow measurements obtained from dynamic computed tomography angiography (CTA) data of AIS patients.

## 2. Methods

### 2.1. Patient vasculature generation

A patient vasculature is created from a mixture of population averages, patient segmentations and scaling laws. We refer to our previous work for a more detailed explanation of the patient vasculature network generation, which for completeness we briefly summarize [13,15,16].

Population averages are taken for the large systemic arteries, starting from the descending aorta up to the circle of Willis (CoW) [12,17]. Different configurations of the CoW can be included in the model, but are not considered in this work. The cerebral vessels beyond the CoW are taken from a dataset of healthy volunteers [18]. At each end of the cerebral vessels, a bifurcating tree is generated using Murray's Law. Murray's Law is given by $R_{i}^{3}=r_{i}^{3}+r_{j}^{3}$ where *R* denotes the mother branch and *r* the daughter branches. The bifurcating trees connect to the pial vessel network by subdivision of the perfusion territories until each outlet is mapped to a single region. For more details, we refer the reader to [13,16,19,20].

The pial vessel network is generated by taking the dual graph of a uniform triangulated surface mesh of the brain. The brain mesh was post-processed using a publicly available model [21,22]. The density of the surface mesh triangles determines the number of surface vessels. The end nodes of the bifurcating trees are connected to the pial vessel network by iterative division of the relevant perfusion territories. Finally, penetrating arteries are generated on the pial surface and connected to the nearest node of the pial surface network. The penetrating arteries perfuse the underlying cerebral tissue and are represented by the model outlets in the blood flow model.

**Figure 1.**
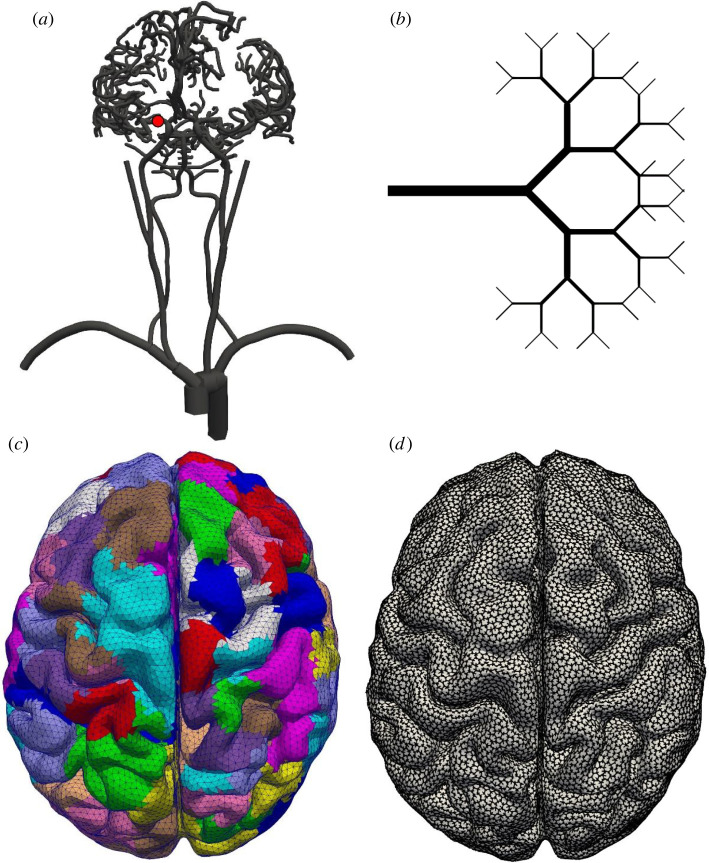
The cerebral blood flow model. (*a*) The major vessels in the model, starting at the heart and ending close to the pial surface, front view. The red dot shows the location of the thrombus used in this paper. (*b*) A bifurcating tree is used to connect the major vessels and the pial surface network. (*c*) Outlet regions on the surface of the brain, top view. Each bifurcating tree covers one outlet surface region. These regions are subdivided at every bifurcation of the bifurcating tree. (*d*) The pial surface vessels form a hexagonal mesh covering the surface of the brain, top view. Penetrating arteries perfuse the underlying cerebral tissue. These penetrating arteries are the outlets of the model on the pial surface.

**Figure 2.**
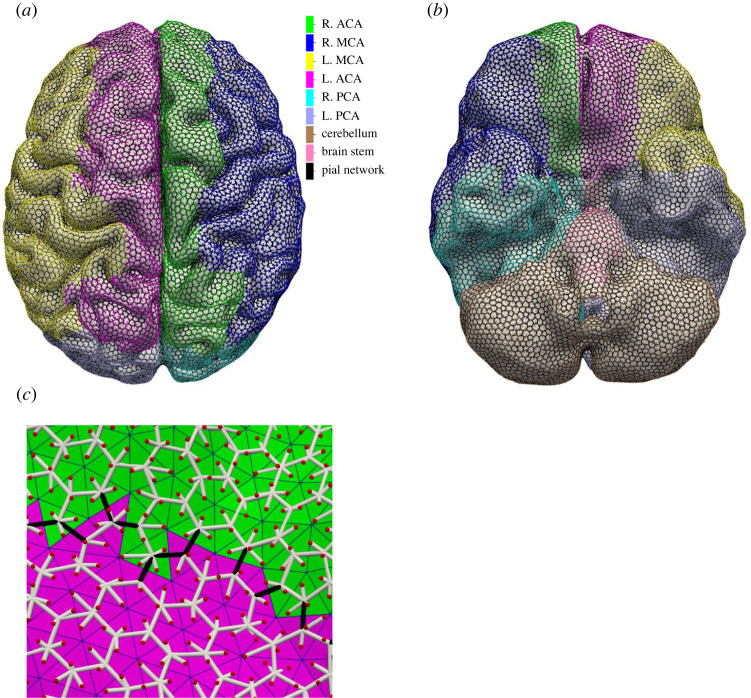
Pial vessel network, top (*a*) and bottom (*b*) views. Vessels are colour-coded based on their perfusion territory. The major regions of the brain are the middle, posterior and anterior regions, on both the left- and the right-hand side, the cerebellum and the brain stem. The pial network vessels cover the surface. (*c*) Close-up of the pial vessel network, the leptomeningeal collateral vessels are shown in black, other vessels in white. The red dots indicate the model outlets on the pial surface and represent the start of the penetrating arteries.

Figure 1 shows the various parts of the patient vasculature generation. The leptomeningeal collateral vessels are defined as the vessels of the pial vessel network that cross the major cerebral perfusion territories, i.e. the major branches of the CoW (M1), cerebellum and the brain stem, see also figure 2. These major perfusion territories have been identified based on vascular atlases obtained from arterial spin labelling perfusion MRI as described in [23–25]. The variations in collateral flow are modelled by including only subsets of the leptomeningeal collateral vessels. A collateral score in the model is defined as the probability (or fraction) of the leptomeningeal collateral vessels that are included in the patient vasculature. The included collateral vessels are chosen randomly from the set of all possible collateral vessels. Since the cerebral surface is built using equally sized triangles, this results in a uniform density (on average) of collateral vessels between all connected regions.

The collateral score in the model is defined as the probability, or fraction, of possible collateral vessels and runs from zero to one. The collateral score used to grade the collaterals for AIS patients clinically is categorical and given as absent, poor, moderate and good [6,7]. To match the collateral score of the model and the clinical score, we estimate the infarct volume in the model and try to match to observations of infarct volume of AIS patients.

### 2.2. One-dimensional blood flow modelling

Blood is modelled as an incompressible fluid while vessels are modelled as elastic tubes. A one-dimensional blood flow model is used to simulate blood flow and pressure during healthy and stroke scenarios. Blood flow is considered to be at steady state, i.e. variables are considered the average over the duration of a heartbeat. Each vessel is discretized with a minimum of three nodes and a maximum grid size of 2.5 mm along its length.

The pressure in the network is calculated by solving the mass balance equation given by

2.1 $$\begin{array}{c} \underset{j}{\sum }G_{ij}(P_{i}-P_{j})=Q_{i} \end{array},$$

where *G_ij_* is the conductance in m^3^ (Pa s)^−1^ (reciprocal of resistance) between nodes *i* and *j*, *P_i_* is the pressure and *Q_i_* is a source term. The resulting system can be written as

2.2 $$\begin{array}{c} GP=\left[\begin{array}{cccc} \sum G_{1j} & -G_{12} & \cdots & -G_{1N}\\ -G_{21} & \sum G_{2j} & \cdots & -G_{2N}\\ \vdots & \vdots & \ddots & \vdots \\ -G_{N1} & \cdots & \cdots & \sum G_{Nj} \end{array}\right]\left[\begin{array}{c} P_{i}\\ \vdots \\ \vdots \\ P_{N} \end{array}\right]=\left[\begin{array}{c} S_{i}\\ \vdots \\ \vdots \\ S_{N} \end{array}\right]=Q \end{array}.$$

The conductance of a segment is given by

2.3 $$\begin{array}{c} G=\frac{\pi r^{4}}{2(\zeta +2)\mu L} \end{array},$$

where *r* is the radius, *L* is the segment length, *μ* is the dynamic viscosity and *ζ* is a dimensionless constant related to the velocity profile, with 2 representing a parabolic profile, i.e. laminar flow, and 9 representing a flatter profile used in this model [26]. The larger constant is the result of a blunt velocity profile in the vessels [27]. It is not exactly known how the details of the velocity profile changes throughout the circulation. A blunt profile is still a good description in arterioles [28].

A pressure–area relationship is used to model the elasticity of the vessel, given by [29]

2.4 $$\begin{array}{c} P=P_{0}+\frac{Eh}{r_{0}^{2}(1-\nu^{2})}(r-r_{0}) \end{array},$$

where *r*_0_ is the initial radius, *P* is the pressure, *P*_0_ is a reference pressure set to the diastolic pressure, *E* is the Young's modulus, *h* is the wall thickness, and *ν* = 0.5 is the Poisson ratio of the vessel wall. The corresponding distensibility for this model is given by

2.5 $$\begin{array}{c} D=\frac{dA}{dP}\frac{1}{A}=\frac{A_{0}2(1-\nu^{2})}{\sqrt{A}\sqrt{\pi }Eh}=\frac{r_{0}^{2}2(1-\nu^{2})}{rEh} \end{array},$$

with values on the order of 10^−6^ Pa^−1^ or 10^−3^ mmHg^−1^, similar to clinical measurements [30].

Model variables for the large systemic arteries (i.e. from the aorta to the CoW) can be found in the appendix and are adapted from [12,17]. The Young's modulus for all remaining cerebral vessels is set to 1.6 MPa.

The wall thickness is calculated as a function of the radius, given by

2.6 $$\begin{array}{c} h=r_{0}(a\exp(br_{0})+c\exp(dr_{0})) \end{array},$$

where *r*_0_ is the initial radius, *a* = 0.2802, *b* = −0.5053 mm^−1^, *c* = 0.1324 and *d* = −0.01114 mm^−1^ [31,32]. This equation results from a curve fit of measurement data on vessel radius and wall thickness in humans.

The system is solved iteratively where the pressure is updated using equation (2.2) and the conductance calculated with the radii from the previous iteration. The radius is then updated using the pressure–area relationship, equation (2.4). Equation (2.2) is solved using lower–upper (LU) decomposition, a direct method. The iterations, solving equations (2.2)–(2.4), are terminated when a pressure tolerance of less than 10^−6^ is reached, defined as

2.7 $$\begin{array}{c} \varepsilon_{P}=\frac{\left|P_{i}-P_{i-1}\right|}{\left|P_{i}\right|}<10^{-6} \end{array},$$

with *P_i_* and *P_i_*_−1_ being the pressure vectors during the *i*th and (i–1)th iterations, respectively. The volume flow rate in a segment, *Q_ij_*, is calculated as

2.8 $$\begin{array}{c} Q_{ij}=G_{ij}(P_{i}-P_{j}) \end{array}.$$

The velocity, *v_ij_*, is calculated as

2.9 $$\begin{array}{c} v_{ij}=\frac{Q_{ij}}{\pi {\left(0.5r_{i}+0.5r_{j}\right)}^{2}} \end{array},$$

where *r* is the radius of the vessel nodes.

At the inlet of the patient network, i.e. the heart, a flow rate and pressure are set. The source terms, *Q_i_*, are set to zero except for the inlet. A known pressure value, *p*_out_, is used at the outlets. Furthermore, the resistance of the outlets is scaled to simultaneously satisfy both boundary conditions (pressure and flow rate). This scaling occurs before an autoregulation step, detailed below, and accounts for the (added) resistance of the vessel in the network. The system is solved until a tolerance of 10^−6^ is reached, given by

2.10 $$\begin{array}{c} \left|\frac{\;p_{0}}{\;p_{\mathrm{in}}}-1\right|<10^{-6},\;\left|\frac{Q_{0}}{Q_{\mathrm{in}}}-1\right|<10^{-6} \end{array},$$

where *p*_0_ and *Q*_0_ are the simulated pressure and flow rate values at the inlet node. Note that after each change, the entire system (equations (2.2) and (2.4)) is solved again until equation (2.7) is satisfied. The fraction between simulated values (*p*_0_, *Q*_0_) and the target values (*p*_in_, *Q*_in_) is used to uniformly scale all outlet resistances.

Autoregulation is an important effect to consider. Autoregulation is the process by which the brain can maintain constant perfusion [33]. The effect of autoregulation is modelled as a change in outlet resistance as a consequence of a drop in perfusion. The resistance at each outlet is free to change between a lower and upper limit. The limits are determined by the maximum dilation or maximum constriction of the downstream vessels.

At the outlets on the pial surface, the resistance is varied to capture the effect of autoregulation. The resistance of the outlets is calculated to achieve a desired volumetric flow rate. The resistance is updated according to

2.11 $$\begin{array}{c} R=\frac{\left(P_{i}-P_{\mathrm{out}}\right)}{\left(Q_{\mathrm{brain}}/N_{\mathrm{total}}\right)} \end{array}.$$

There are limits to the range of autoregulation. The outlet resistance is limited by

2.12 $$\begin{array}{c} R_{\mathrm{low}}=\frac{P_{\mathrm{low}}}{\left(Q_{\mathrm{brain}}/N_{\mathrm{total}}\right)},\;R_{\mathrm{upp}}=\frac{P_{\mathrm{upp}}}{\left(Q_{\mathrm{brain}}/N_{\mathrm{total}}\right)} \end{array},$$

where *P*_low_ and *P*_upp_ set the pressure limits at which the downstream tissue can no longer regulate flow and *N*_total_ is the number of outlets on the pial surface. The outlet resistance is limited between the lower and upper bounds. *P*_low_, *P*_upp_ and *Q*_brain_ are set to 10 mmHg, 100 mmHg and 12.5 ml s^−1^, respectively [34,35]. The system is solved again using LU decomposition with the updated resistance, and is iterated until a tolerance of 10^−6^ is reached, defined as

2.13 $$\begin{array}{c} \varepsilon_{R}=\frac{{\left|R_{i}-R_{i-1}\right|}_{\infty }}{{\left|R_{i}\right|}_{\infty }}<10^{-6} \end{array},$$

with ***R_i_*** being the outlet resistance vector during the *i*th iteration and ***R_i−1_*** being the outlet resistance vector from the (*i* − 1)th iteration.

A permeable thrombus is modelled using Darcy's Law for porous media. Darcy's Law, integrated over a vessel segment in one dimension, is given by

2.14 $$\begin{array}{c} Q=\frac{\kappa A}{\mu L}\Delta p=G\Delta p=\frac{\Delta p}{R} \end{array}.$$

The total resistance of a vessel segment containing a thrombus is the sum of the vessel resistance and the thrombus resistance. The vessel resistance is generally small compared with the thrombus resistance. Thrombus resistance is the dominant term when thrombus permeability is low. The total resistance of the vessel segment containing a thrombus is given by

2.15 $$\begin{array}{c} R_{T}=R_{\mathrm{thrombus}}+R_{\mathrm{vessel}} \end{array},$$

where *R*_vessel_ is given by equation (2.3), and *R*_thrombus_ is given by equation (2.14).

The infarct volume is calculated as

2.16 $$\begin{array}{c} IV=\frac{V_{\mathrm{brain}}}{N_{\mathrm{total}}}N_{\mathrm{outlets}} \end{array},$$

where *V*_brain_ is the brain volume, *N*_total_ is the total number of outlets at the pial surface and *N*_outlets_ is the number of outlets on the pial surface where the change in flow rate is below a threshold. The threshold chosen in this paper is 0.4, i.e. 60% relative flow rate compared with a healthy simulation [36–39]. This threshold is commonly used to estimate the penumbra i.e. infarct region without treatment. Since treatment and tissue infarction are not modelled, this threshold is chosen to capture the final infarct volume. This threshold is not time dependent, making it a good choice for the model [40]. The change in flow rate is defined as a fraction (fractional flow rate change), given by

2.17 $$\begin{array}{c} \Delta Q=\frac{Q_{\mathrm{healthy}}-Q_{\mathrm{stroke}}}{Q_{\mathrm{healthy}}} \end{array}.$$

Table 1 lists the values of the parameters used in the model.

**Table 1.** Model parameters.

parameter	value	unit
*p*_in_	12 500	Pa
*p*_out_	666	Pa
*Q*_in_	100	ml s^−1^
*ν*	0.5	–
µ	3.5	mPa
*ζ*	9	–
length–radius ratio	10	–
Murray's exponent	3	–
pial vessel radius	0.2	mm
*V*_brain_	1390	ml
*N*_total_	113 913	–
penetrating artery density	1	mm^−1^
*P*_low_	10	mmHg
*P*_upp_	100	mmHg
*Q*_brain_	12.5	ml s^−1^

Most of the computational cost of the model is associated with the autoregulation step, resulting in many iterations. All simulations are performed on a standard desktop computer with an Intel core i7-7700 k running at 4.2 GHz with 16 GB RAM. An average simulation has about 600 k nodes in 180 k individual vessels. Resulting in a run time of around three hours per simulation on a single core. The source code is available at https://github.com/Rpadmos/Collaterals_Porous-clots.

### 2.3. Measurements in acute ischaemic stroke patients

The transit time over the occlusion, thrombus length and void fraction have previously been measured using dynamic CTA data of 44 AIS patients from the Amsterdam University Medical Center and the Dutch acute stroke trial (DUST) [41]. These patients had a single large vessel occlusion in the middle cerebral artery (MCA). From the dynamic CTA data, contrast arrival along the occluded vessel was observed by measuring time attenuation curves.

The transit time between the beginning and the end of the occlusion was computed using a cross-correlation of the time attenuation curves. The thrombus void fraction was calculated as the ratio between the mean contrast intensities within the thrombus and the contralateral non-occluded vessel [14]. Thrombus length was calculated as the distance between adjacently placed markers along the occlusion. More details can be found in [41].

The patient measurements are used to calculate the flow rate through the segment, given by

2.18 $$\begin{array}{c} Q=\frac{\epsilon A_{MCA}L}{\Delta t}, \end{array}$$

where ɛ is the void fraction, *L* is the thrombus length and Δ*t* is the transit time. The flow rate *Q* is used in the model to determine the resistance for each collateral score. The resistance values are then used to estimate the pressure drop, thrombus permeability and infarct volume for each patient.

## 3. Results

**Figure 3.**
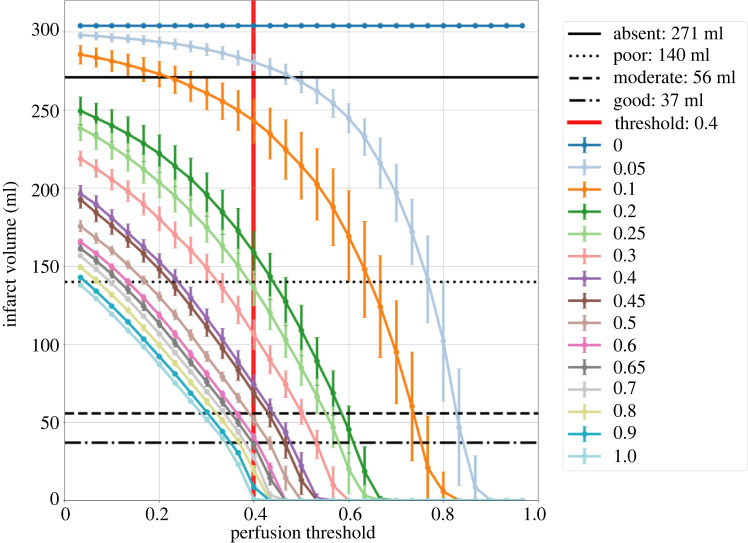
The estimated infarct volume for a range of collateral scores, from zero to one as a function of the perfusion threshold. The thrombus is located in the right MCA and completely impermeable. The four black lines show the median final infarct volumes as measured for AIS patients for different collateral scores [6]. The red vertical line shows the threshold at 0.4, i.e. 60% relative flow rate compared with the healthy simulation. Error bars show the standard deviation over five simulations.

Figure 3 shows a plot of the infarct volume estimated using the one-dimensional BF model as a function of the perfusion threshold, and the median final infarct volume as measured in AIS patients [6]. The values of 0.05, 0.25, 0.5, 0.65 for the collateral vessel probability are chosen as the values that will represent the absent, poor, moderate and good collaterals (table 2). Variation within these groups can be simulated by varying the model parameters around these values.

**Table 2.** Patient measurements (taken from [6]) and model estimates with the perfusion threshold at 0.4.

collateral score	number of patients	final infarct volume median ml (IQR)	model mean infarct volume (ml)
absent	20	271.0 (105.8–411.8)	280.5
poor	126	139.8 (63.9–228.1)	136.0
moderate	180	55.9 (27.4–107.8)	51.7
good	123	36.9 (15.4–87.6)	35.8

**Figure 4.**
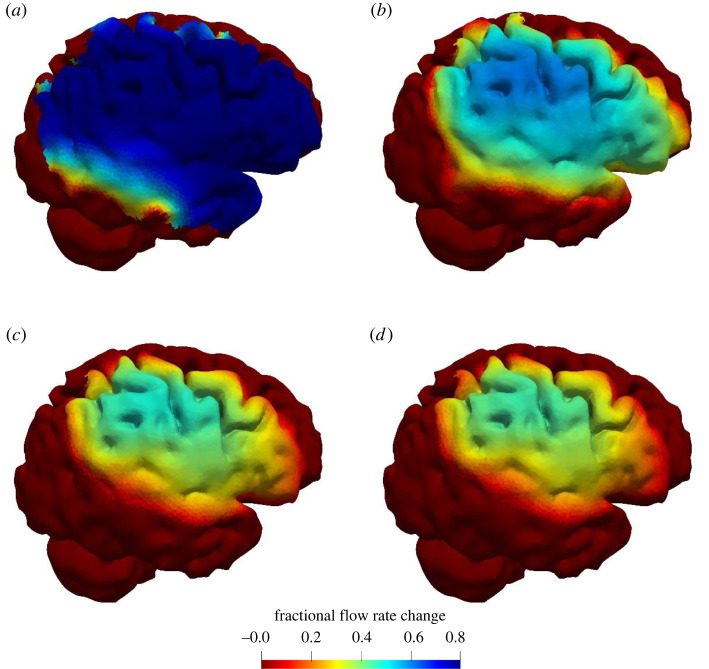
Fractional flow rate change on the pial surface during an AIS for the various collateral scores. Collateral scores: (*a*) absent, (*b*) poor, (*c*) moderate and (*d*) good. Side view of the brain showing the right hemisphere. The thrombus is located in the right MCA, and completely occluding the vessel.

The thrombus is located in the right MCA. During the healthy simulation, the entire brain is perfused at the same flow rate even if the perfusion pressure across the surface is different. Autoregulation ensures that the volumetric flow rate to the cerebral tissue is constant. During AIS, autoregulation at the outlets compensates for the reduction in perfusion by lowering the resistance, i.e. vessel dilation. Depending on the collaterals, the effects of the thrombus can be compensated for by the reduction in resistance. The fractional flow rate change on the pial surface during an AIS for the various collateral scores is shown in figure 4.

**Figure 5.**
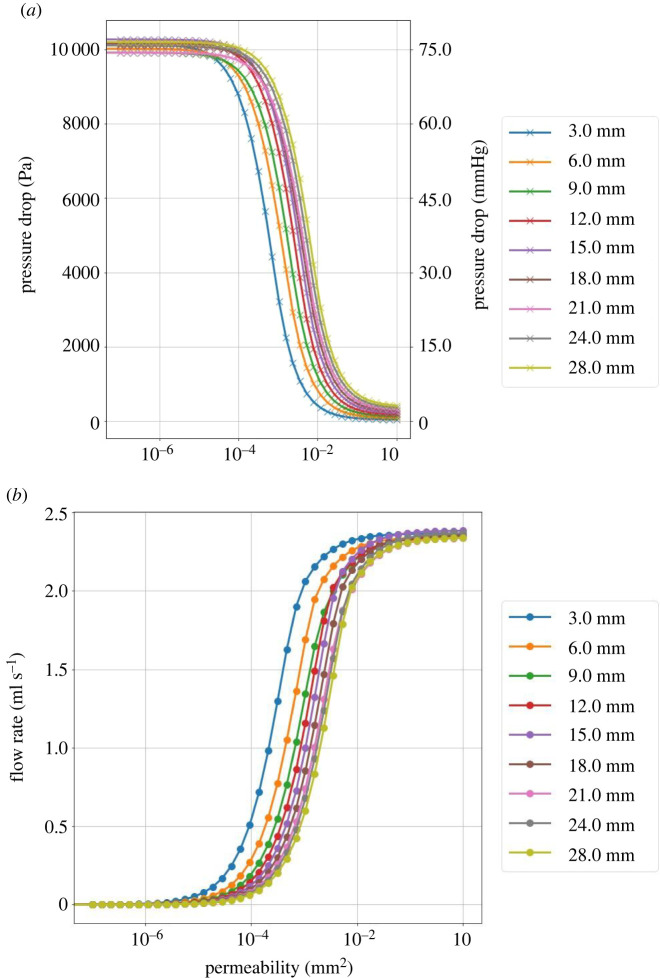
(*a*) Pressure drop over the thrombus as a function of permeability for various thrombus lengths. (*b*) Flow rate through the thrombus as functions of permeability for various thrombus lengths. The collateral score is set to 0.05 (absent) for these simulations. Plots for the different collateral scores are similar. The pial vessel network is different, i.e. randomly generated, for each thrombus length.

Figure 5 shows the pressure drop over the thrombus and the flow rate through the thrombus as a function of the thrombus permeability for absent collaterals. Different lengths of thrombi were considered. As the permeability increases, the flow rate through the thrombus increases and the pressure drop decreases. For thrombi with high permeability, the flow reaches a maximum (i.e. healthy value). Thrombi are typically between a few mm and a few cm [41]. Various thrombi lengths, ranging from 3 to 28 mm are therefore simulated.

**Figure 6.**
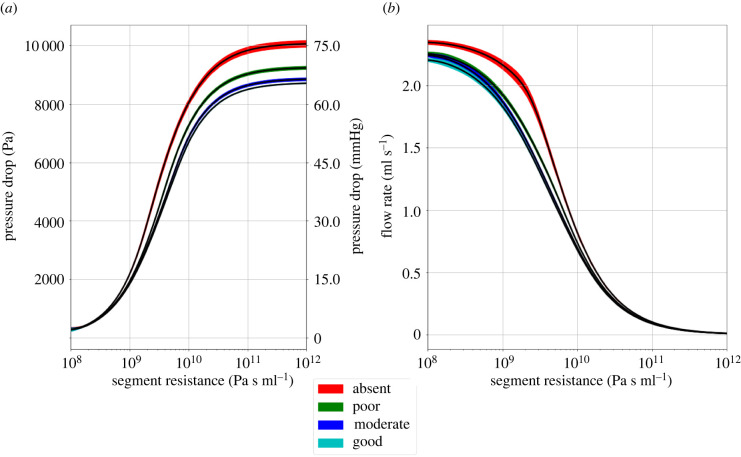
(*a*) Pressure drop over the thrombus as a function of the segment resistance. (*b*) Flow rate through the thrombus as a function of the segment resistance. The different colours indicate the collateral score: absent, poor, moderate and good. The filled areas around the curves show the standard deviation over nine simulations, where the thrombus length is varied as shown in figure 5.

Figure 6 shows the pressure drop over the thrombus and the flow rate through the thrombus as a function of the segment resistance, given by equation (2.15), and collateral score. There is a difference in the distal pressure between the collateral scores. For low-resistance thrombi, the flow rate through the thrombus is different as a result of the compensatory effect of the collaterals.

**Figure 7.**
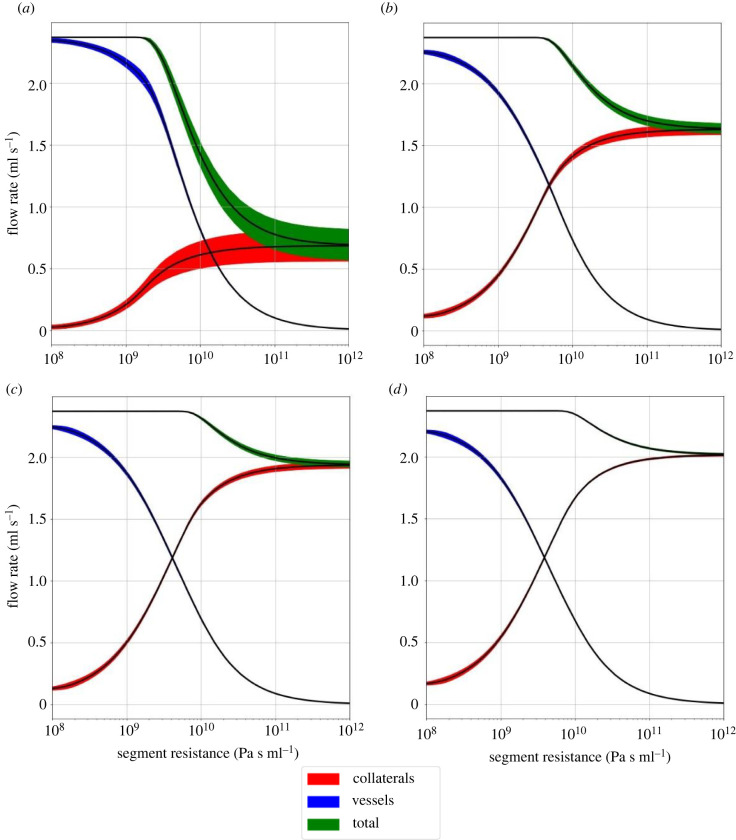
Flow rates to the territory of the right MCA during an AIS as a function of the segment resistance for the various collateral scores. Collateral score: (*a*) absent, (*b*) poor, (*c*) moderate and (*d*) good. The total flow to the region is shown in green, which is the sum of the flow through the thrombus (blue), and the collateral circulation (red). The filled areas around the curves show the standard deviation over nine simulations, where the thrombus length is varied as shown in figure 5.

Figure 7 shows the total flow rate to the distal tissue of the affected region. The total flow is the sum of the flow through the thrombus and the collaterals. Autoregulation is able to maintain adequate perfusion up to a threshold, set by the limits defined in the model. As the resistance of the occluded segment increases, the collaterals become more important. For high-resistance thrombi, the remaining flow to the distal tissue is entirely due to the collaterals.

**Figure 8.**
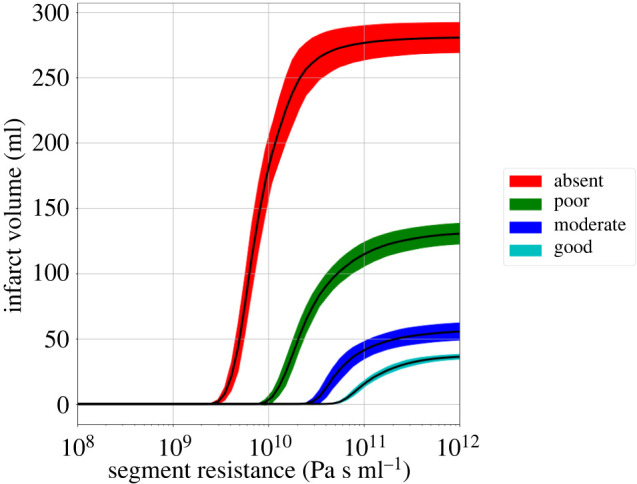
Estimated infarct volume as a function of segment resistance for the various collateral scores. The different colours indicate the collateral score: absent, poor, moderate and good. The filled areas around the curves show the standard deviation over nine simulations, where the thrombus length is varied as shown in figure 5.

Figure 8 shows the estimated infarct volume as a function of resistance and the collateral score. The maximum infarct volume changes significantly depending on the collateral score, with some variation due to the spatial location of the collateral vessels on the pial surface. Better collaterals are able to compensate for more impermeable thrombi.

**Figure 9.**
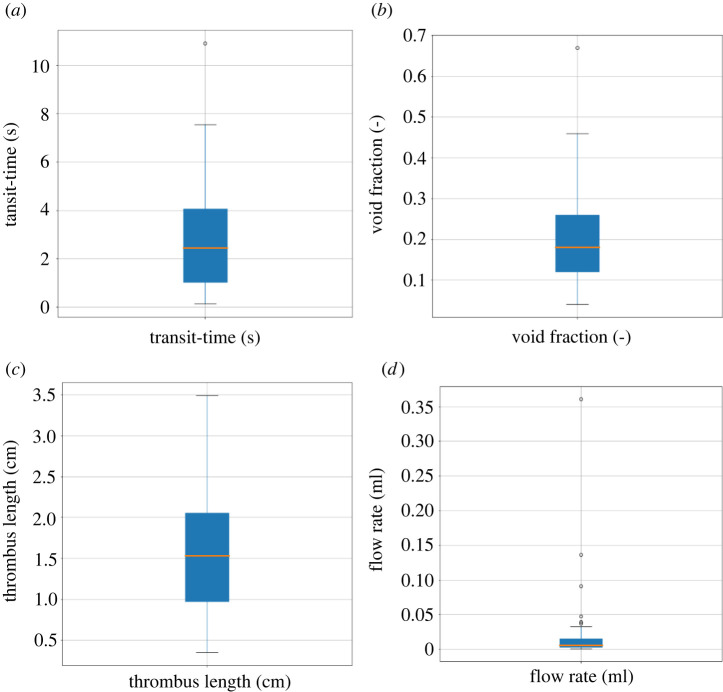
Box plots of the measurements for 44 AIS patients. (*a*) transit times, (*b*) void fractions, (*c*) thrombus lengths and (*d*) flow rates.

**Figure 10.**
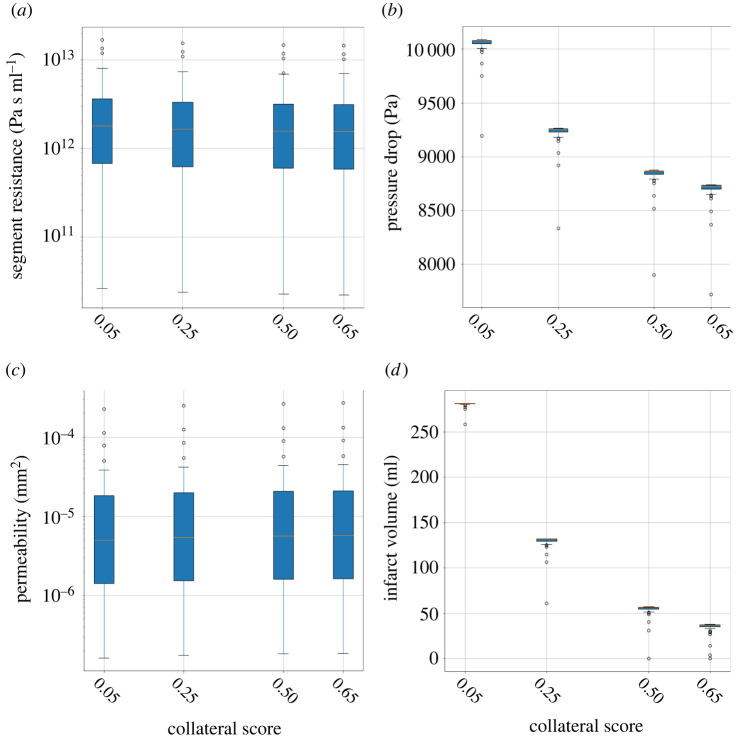
Estimates derived from the one-dimensional BF model using the patient measurements (thrombus length and flow rate) for the collateral scores: absent, poor, moderate and good. (*a*) segment resistance, (*b*) pressure drop over the thrombus, (*c*) thrombus permeability and (*d*) infarct volume.

Figure 9 shows box plots of the time delay, void fraction, thrombus length and flow rate for 44 AIS patients. Figure 10 shows box plots of the estimated resistance, pressure drop, thrombus permeability and infarct volume for the 44 AIS patients for each collateral score. The segment resistance and thrombus permeability vary over more than three orders of magnitude, but the resulting flow rate is relatively small, corresponding to less than 1% of a non-occluded vessel (compared with a healthy simulation), the effect on infarct volume is therefore small. The pressure drop over the thrombus and the estimated infarct volume are mostly a result of the collaterals with a small contribution of the thrombus permeability.

## 4. Discussion

The blood flow model considered in this work consists of a network of (variable) resistances. Thrombi considered in this work are assumed to be completely occluding the vessel with homogeneous permeability. *In vivo* thrombi are very heterogeneous in their structure and composition [42]. The permeability reported in this work should be considered to be an effective permeability of the thrombus. For instance, a thrombus that is completely impermeable but has a number of micro channels, or a severe stenosis could produce the same effect as a permeable thrombus in this model. Their effect on the pressure drop and flow rate can be captured by an effective permeability, or equivalently a thrombus resistance.

The collateral score in the model is varied by considering the number of possible connections on the surface, as shown in figure 3. What matters most for the collateral flow is the conductance between the different regions. As each collateral vessel has the same size, varying their number results in a scaling of the conductance between regions. The same conductance between regions can be achieved by fewer but larger vessels. Unfortunately, there is not much known about the collateral circulation in humans. As a result, the model is inevitably full of assumptions that will need to be tested in the future. The collateral density varies significantly between species and individuals [43]. The model presented here captures the effect of variation in collateral density and its effect during acute ischaemic stroke. In addition, the collateral vessels are resampled for each simulation to capture the variability in their location on the surface.

Figure 4 shows the simulated fractional flow rate change during an AIS for the collateral scores. The thrombus is located in the right middle cerebral artery, a common thrombus location, and completely occludes this vessel [44]. Occlusion of this vessel leads to a drop in perfusion throughout its perfusion territory. Depending on the thrombus permeability and collateral score, the affected tissue will still receive blood flow. The collaterals of the one-dimensional BF model are calibrated to obtain similar infarct volumes as for the various collaterals scores. Variation in the collaterals within each class can capture some of the variation in infarct volume. The infarct volume considered in the model is defined as the volume below a certain threshold, 40% flow rate drop or more, which seems to be a realistic threshold to define the boundary of tissue at risk (penumbra) [36]. In the future, we will replace this threshold with a mechanistic model of infarct formation [45].

The final infarct volume in AIS patients is often determined on follow-up scans days after treatment. Infarct volume in the model can therefore be considered the maximum possible infarct volume, essentially the penumbra and core volume on stroke onset. Variation in the total brain volume between individuals probably contributes to the spread in observed infarct volumes. The maximum infarct volume for the right MCA for the mesh used in this study is 309 ml. The data used to calibrate the collateral parameter also contains other thrombus locations, making a direct comparison difficult [6]. The model is therefore calibrated to the median infarct volume for each collateral score as shown in figure 3. Choosing the median value does lead to a non-zero model value for the absent collaterals and non-zero collateral flow as shown in figures 3 and 7, respectively.

Firstly, we look at the pressure drop and flow rate through the thrombus as a function of the collaterals and thrombus permeability. Figure 5 shows the pressure drop over the thrombus and the flow rate through it as a function of the thrombus permeability. The flow through the thrombus and the pressure drop show an s-shaped curve, i.e. sigmoid function. Figure 6 shows the pressure drop over the thrombus and the flow rate through it as a function of the thrombus resistance. The results of the different simulations all collapse to a single curve for a constant collateral score. The thrombus resistance captures the effect of the length and permeability of the thrombus. A short and impermeable thrombus can lead to the same pressure drop and flow rate as a long but permeable thrombus. The collaterals, in addition to providing residual flow to the affected tissue, increase the pressure distally of the thrombus, lowering the pressure drop.

Most of the resistance of the vasculature is due to microcirculation [46]. This means that for a thrombus to significantly affect the flow through the vessel, its resistance has to be of comparable magnitude. Based on the flow rates shown in figure 7, this seems to be around 10^9^ Pa s ml^−1^. For a high-resistance thrombus, changes in the pressure distally of the thrombus do not significantly affect the flow through it. The change in pressure is simply too small to overcome the high resistance of the thrombus. For low resistance, the distal pressure due to the collaterals does not affect the flow much either, and the flow returns to its healthy value.

Figure 7 shows the perfusion to the tissue at risk, i.e. the right MCA territory, during an AIS as a function of the thrombus resistance and collateral score. The variability of the collateral vessels seems to decrease with increasing collateral score. This probably reflects the variability in collateral vessel location which is more variable with lower density, i.e. collateral score. The collateral vessels compensate for the change in thrombus resistance when the drop in perfusion is not severe. Eventually, the vessels are maximally dilated, i.e. the resistance is minimal, and flow from the surrounding tissue is maximal. The degree of remaining flow is based on the degree of collateral vessels. Without autoregulation, there is an immediate drop in perfusion when there is a thrombus. The pressure drop across the thrombus becomes smaller as the distal pressure increases.

The aim of this model is to capture the effect of the collaterals and thrombus permeability on cerebral blood flow during AIS. The model ends at the penetrating arteries. Arteries beyond this point can be considered to be part of the microcirculation. Modelling the microcirculation requires accounting for the cellular nature of blood [47].

For different collateral scores, a range of thrombus permeability and lengths are simulated. We find that the thrombus permeability ranges between 10^−7^ and 10^−4^ mm^2^ (figure 10). *In vitro* estimates of the permeability range from 10^−11^ to 10^−7^ mm^2^ [48]. These estimates differ by several orders of magnitude. These *in vitro* thrombi are composed purely of fibrin and platelets, without any red blood cells or other components, potentially leading to very dense thrombi. The measurements of AIS patients only include cases that show some contrast distal to the occlusion (either due to in-thrombus flow or collateral retrograde flow) [41]. These thrombi might only partially occlude the vessel, leading to an effective permeability that is much higher than the estimates obtained from *in vitro* thrombi experiments.

The pressure drop over the thrombus has been measured *in vivo* [49,50]. The observed pressure drop was found to be 56.7 ± 18.0 mmHg for patients with a modified Rankin scale (mRS) of 0–2 and 63.1 ± 19.1 mmHg for patients with a mRS of 3–6 [49]. The median values predicted by the one-dimensional BF model, as shown in figure 10, range between 65.1 and 75.3 mmHg. The pressure drop depends largely on the collaterals, as well as (inlet) blood pressure. Nevertheless, the values obtained from the model are similar to those found in the literature. Unfortunately, the lack of model validation using more direct comparison currently remains a limitation.

The collaterals are a dominant factor in determining the infarct volume, as well as the pressure drop across the thrombus. The collaterals also play an important role during thrombolysis as it can allow the thrombolytic agent to reach both ends of the thrombus, potentially dissolving it from both ends. A permeable thrombus could affect thrombolysis in various ways. Firstly, a permeable thrombus would allow flow to quickly reach the thrombus, significantly speeding up thrombolysis, and leading to successful treatment [9]. Without permeation, thrombolysis would be diffusion-limited and require hundreds of minutes [10]. In addition, the permeable thrombus would also allow the thrombolytic agent to reach inside the thrombus, dissolving it from inside. Estimating thrombus permeability could allow clinicians to predict how effective thrombolytic treatment would be.

## 5. Conclusion

In this paper, we modelled the leptomeningeal collateral circulation and thrombus permeability during an acute ischaemic stroke. Residual blood flow to the tissue is maintained by flow through the occluded vessel and by the collaterals. Having good collaterals and a permeable thrombus both contribute to positive patient outcome. The pressure drop over the thrombus depends on the resistance (permeability) of the thrombus and the collaterals. Good collaterals decrease the pressure drop over the occlusion by increasing the distal pressure. At the same time, a higher permeability results in lower resistance of the thrombus, and leads to a smaller pressure drop. Thrombus permeabilities were estimated for AIS patients for various collateral scores, and found to be between 10^−7^ and 10^−4^ mm^2^ for permeable thrombi. Our simulations suggest that the collaterals are a dominant factor in determining the infarct volume during a stroke.

## Data Availability

The source code is available at https://github.com/Rpadmos/Collaterals_Porous-clots.

## Appendix A

Table 3.

**Table 3.** Vessel parameters. Adapted from [12,17].

ID	vessel name	length (mm)	radius (mm)	Young’s modulus (MPa)
1	ascending aorta	40	12	0.4
2	aortic arch I	20	11.2	0.4
3	brachiocephalic	34	6.2	0.4
4	aortic Arch II	39	11	0.4
5	L. common carotid	208	2.5	0.4
6	R. common carotid	177	2.5	0.4
7	R. subclavian	34	4.23	0.4
8	thoracic aorta	156	9.99	0.4
9	L. subclavian	34	4.23	0.4
10	L ext. carotid	177	1.5	0.8
11	L int. carotid I	177	2	0.8
12	R int. carotid I	177	2	0.8
13	R ext. carotid	177	1.5	0.8
14	R. vertebral	148	1.36	0.8
15	R. brachial	422	4.03	0.4
16	L. brachial	422	4.03	0.4
17	L. vertebral	148	1.36	0.8
18	L. PCoA	15	0.73	1.6
19	R. PCoA	15	0.73	1.6
20	basilar I	5.6	1.62	1.6
21	L. MCA	30	1.43	1.6
22	R. MCA	30	1.43	1.6
23	L. ACA, A1	12	1.17	1.6
24	R. ACA, A1	12	1.17	1.6
25	L. PCA, P1	5	1.07	1.6
26	R. PCA, P1	5	1.07	1.6
27	L. ACA, A2	13.96	1.2	1.6
28	R. ACA, A2	13.96	1.2	1.6
29	ACoA	3	0.74	1.6
30	L. PCA, P2	6.59	1.05	1.6
31	R. PCA, P2	6.59	1.05	1.6
32	R. SCA	10	0.78	1.6
33	L. SCA	10	0.78	1.6
34	R. AICA	10	0.63	1.6
35	L. AICA	10	0.63	1.6
36	basilar II	5.6	1.62	1.6
37	pontine I	5	0.2	1.6
38	pontine II	5	0.2	1.6
39	pontine III	5	0.2	1.6
40	pontine IV	5	0.2	1.6
41	pontine V	5	0.2	1.6
42	pontine VI	5	0.2	1.6
43	pontine VII	5	0.2	1.6
44	pontine VIII	5	0.2	1.6
45	pontine IX	5	0.2	1.6
46	pontine X	5	0.2	1.6
47	pontine XI	5	0.2	1.6
48	pontine XII	5	0.2	1.6
49	basilar III	5.6	1.62	1.6
50	basilar IV	5.6	1.62	1.6
51	basilar V	5.6	1.62	1.6
52	R. PICA	10	0.63	1.6
53	L. PICA	10	0.63	1.6
54	R. vertebral II	20	1.36	0.8
55	L. vertebral II	20	1.36	0.8
